# Novel study designs to investigate the placebo response

**DOI:** 10.1186/1471-2288-11-90

**Published:** 2011-06-10

**Authors:** Paul Enck, Sibylle Klosterhalfen, Stephan Zipfel

**Affiliations:** 1 University Hospital Tübingen, Dept. of Psychosomatic Medicine, Tübingen, Germany; 2 University of Düsseldorf, Institute for Clinical Neurobiology and Medical Psychology, Düsseldorf, Germany

**Keywords:** study design, experimental research, placebo

## Abstract

**Background:**

Investigating the size and mechanisms of the placebo response in clinical trials have relied on experimental procedures that simulate the double-blind randomized placebo-controlled design. However, as the conventional design is thought to elucidate drug rather than placebo actions, different methodological procedures are needed for the placebo response.

**Methods:**

We reviewed the respective literature for trials designs that may be used to elucidate the size of the placebo response and the mechanisms associated with it.

**Results:**

In general, this can be done by either manipulation the information provided to the subjects, or by manipulation the timing of the drug applied. Two examples of each strategy are discussed: the "balanced placebo design" (BDP) and the "balanced cross-over design" (BCD) and their variants are based on false information, while the "hidden treatment" (HT) and the ""delayed response test" (DRT) are based on manipulating the time of drug action. Since most such approaches include deception or incomplete information of the subjects they are suitable for patient only with authorized deception.

**Conclusion:**

Both manipulating the information provided to subjects (BDP, DCD) or manipulating the timing of drug application (HT, DRT) allows overcoming some of the restrictions of conventional drug trials in the assessment of the placebo response, but they are feasible mostly in healthy subjects for ethical reasons.

## Review

### Placebo response and placebo effect

Ever since the dawn of the first randomized placebo-controlled trials testing new drugs and treatments in the middle of the last century, and even before [1], placebo responses in clinical trials have given rise to discussion and concern regarding their mechanisms, and have usually been regarded as a nuisance or a barrier to a rational approach in modern drug development. High placebo responses have induced false expectations regarding drug efficacy and resulted in the refusal or withdrawal of drugs in some cases, e.g. neurokinins in the treatment of depression.

Not only do placebo responses in clinical trials impose significant limits to the testing of new compounds, but they are also linked to the drug adherence and compliance of patients in such trials in a paradoxical way. Patients that adhered to medication instructions by more than 80% showed better survival in a coronary disease study, and poor drug adherence in a myocardial infarction survivor study was associated with a higher risk of mortality [2] irrespective of whether the active compound or a placebo was taken, and regardless of other potential risk factors. This has been attributed to the greater expectancies or beliefs, both in drug and placebo responders that the medication may be of help, although other factors, such as health behaviors, cannot be ruled out completely. These findings have certainly fostered the development of further experimental approaches to the placebo phenomenon [3].

Attempts to unravel the mechanisms of the placebo response in clinical trials have used meta-analytic approaches of the placebo arm of trials - with mixed results. The drug-placebo difference in randomized controlled trials has been reported to be around 40% in functional disorders [4] but lower in depression (29%), bipolar mania (31%) and migraine (21%) [5,6]. The reasons for these variable placebo effect rates are unknown but may include the sample size [4], the year of study [7], design characteristics [6], and recruitment pattern [8]. Meta-analyses can come to opposite conclusions on the same data set, e.g. with respect to the direction of the effects of the number of study visits on the placebo effect size [9,10], but this may be due to data extraction errors that lead to false findings and conclusions [11]. Other contributing factors to the placebo response rate in clinical trials were: the origin of patients - response rates in migraine prophylaxis were higher in Europeans than in North Americans [6] - personal expectations [12] and the loss thereof, e.g. in Alzheimer's disease [13], the study center [14], and patient recruitment and physician training [8]. A genetic contribution to placebo responsiveness has been shown [15] but strong empirical evidence is still lacking.

Because of the difficulties to reliably identify placebo responders and predicting placebo response rates in clinical and experimental trials, different methodological attempts have been made to the way (novel) drugs are tested against placebo. At the same time, but to a varying degree, these and other designs have also be used to unravel some of the mechanisms behind the placebo response. This review will discuss experimental designs that may allow characterization and quantification the placebo response. We will introduce two distinctly different approaches to elucidate the mechanisms behind this response, i.e. by manipulating the information provided to participants and by manipulating the timing of drug action, and will present examples for both from the current literature.

Throughout this review we will use the following terminology: placebo response is the response observed in patients receiving a placebo treatment in a trial, which is measured by within-group changes. Placebo effect, in contrast, is the effect attributable to the placebo treatment after controlling for other non-specific or extraneous factors such as the natural history and regression to the mean; this is measured by the difference between a placebo group and a no-treatment control group in a trial [16]. Thus, the placebo response is expected to be larger than the placebo effect, and the difference between both is explained by other factors. Our review will not focus on designs for better discrimination between the drug and the placebo effect in clinical trials.

### Traditional designs

The most traditional way to attempt to "control" for placebo response in clinical trials was the use of a crossover design, in which an individual patient serves as her/his own control, reducing the between-subject variability and the number of patients studied. Data from crossover trials can also be used to identify predictors of the placebo response in various clinical conditions.

This model was almost completely abolished due to the fact that blinding may be rather difficult in such studies [17,18]. Although "active placebos" that mimic the side effects of a compound without inducing its main effects have been used to avoid such problem [19], this approach can sometimes introduce bias into trials given that some "active placebos" may not be inert [18]. Another major limitation of the crossover design is the interference of learning effects: prior exposure to drug influences the effects of subsequent treatment and prior exposure to placebo may affect drug efficacy [20,21].

Another conventional model to rule out placebo responses is the use of a placebo run-in phase prior to drug and placebo dispensing to identify and exclude placebo responders: Placebo responders tend to exhibit less severe symptoms during run-in [22-25], and to respond faster to treatment with symptom improvement [26] than patients in the drug arm. Drug-free run-in periods have also been used to identify individual and group characteristics of placebo responders. However, these results cannot be generalized across medical conditions [24,27] since most of the variables that are regularly documented at study initiation are related to symptoms and disease characteristics rather than to individual personality traits or states [28]. Extensions of placebo run-in periods are studies with multiple drug/placebo phases that alternate, with or without washout periods in-between [29]. These models were more recently requested again by drug approval authorities to account for variable symptom courses and the alternation of symptom-free with relapse periods in many chronic diseases. It has, however, been shown that the placebo response in a first medication period does not reliably predict the response (to drug or placebo) in a second phase [30].

If being a placebo responder is a stable characteristic of an individual patient - which some of the published data question - study designs should take this into account by employing a design with multiple (>2) crossovers between placebo and drug, and to randomize and individualize in a "single subject trials" (SST) the timing for run-in and run-out for each phase [31]. In theory, this should allow to reliably distinguish placebo responders from non-responders. However, multiple crossovers with randomly assigned treatment periods, with a complete random order or a random starting day may generate specific methodological problems and need new statistical models before being applicable in clinical drug testing. One such novel clinical model was tested recently in the therapy of Parkinson's Disease called the Delayed-Start Study Design (also named the randomized-start design) [32].

While all of these designs may be able to improve drug testing and the distinction between drug and placebo response, most of these designs are not able to characterize the nature of the placebo response and the mechanisms behind it. Even meta-analyses of the placebo arms of drug trials as discussed above will only allow describing potential predictors of the placebo response that need prospective validation in experimental or clinical settings.

### Experimental designs

In laboratory research, a number of experimental designs have been employed that may help to identify and characterize predictors of the placebo response in the future. They have been generated by theoretical issues such as the "additive model" as discussed by Kirsch 2000 [33]. Some of these models have already been tested in laboratory settings, while others are based on theoretical considerations and wait for their empirical approval. Different from clinical trials, experimental designs allow control over some of the factors that are believed to drive the placebo response, e.g. the wording and timing of information provided [34], the gender and social status of the experimenter and the subject [35], their emotional state [36] and psychological and genetic traits [15,37].

### The problem of the "additive-model" assumption

**Figure 1:**
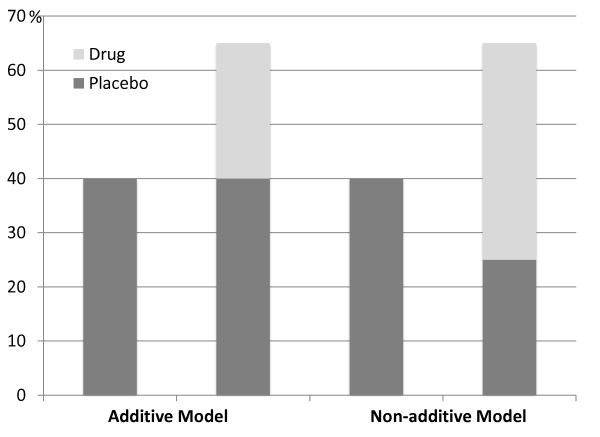
**The "additive model" and the "non-additive model" according to Kirsch (2000): **All placebo-controlled drug trial are currently based on the assumption that the placebo response in the drug arm of the study is equal to the placebo response in the placebo arm; however, it may be either smaller or greater.

Randomized and placebo-controlled clinical trials usually operate under the assumption that drug and placebo effects are additive in the drug arm of the study (Figure 1), thus subtracting the response in the placebo arm of the study from the response in the drug arm would allow to assess the "true" drug efficacy. This assumption is inherent in all current drug trials and is present also in most experimental studies.

This model has, however, been questioned [38]. One argument against the additive model is incomplete blinding with many drugs, so that patients/subjects may be aware of the experimental condition by taking adverse events into account. This would increase the placebo response in the drug group and reduce the placebo response in the placebo group. Recently, neurobiological evidence against the additive model has been provided [39] and is summarized elsewhere [40].

Therefore, Kirsch & Weixel [38] and Kirsch [33] proposed the "balanced placebo design" as an answer to this open question.

### The balanced placebo design (BPD)

The so-called "balanced placebo design" (BPD) was traditionally used in the testing for expectancy effects of frequently consumed everyday-drugs such as caffeine, nicotine and alcohol [41], recently also with drugs such as marijuana [42].

While one half of the study sample receives placebo and the other half the drug, half of each group is receiving correct information while the other half is receiving false information on the nature of their study condition (drug or placebo) immediately prior to drug testing, thus allowing to differentiate between the "true" drug response (those receiving the drug but are told they received placebo) and the true placebo response (those receiving placebo but are told they received the drug).

The "non-additive model" according to Kirsch [33] can be tested in the following way: If the difference between the drug response plus the placebo response and true drug response is unequal to the true placebo response, the non-additive assumption is correct.

A variant of the BPD is the "half BPD" in which all subjects are given placebos, but half of them receive information that they receive the drug - this is the most common design in current placebo research, as it does not require approval for performing a drug study. However, effective double blinding of such as study is difficult unless - as in a recent test in our laboratory - the subjects and the experimenter(s) conducting the study are made to believe that they participate in a full BPD.

As is evident, BPD studies imply deception of the subjects [43]. This limits its suitability and acceptance outside the laboratory and in patients for ethical reasons [44] unless the proposed "authorized deception" is implemented. It has recently been shown in an experimental placebo analgesia trial that authorized deception will not corrupt the data collection [45].

One of the pitfalls of the BPD is the fact that all subjects are informed (either correctly or falsely) prior to testing whether and what they have received. In sceptical subjects (especially medical students), this may raise doubts about the truth of the information provided and may require additional measures, such as a reliable "cover story" about the rational why the information is given at all. This is usually done by informing them that once the drug is active, the information whether and what they received may no longer be relevant - however, the subjects' acceptance of such information is difficult to prove prior to the test, and its testing afterwards may be subject to another bias.

### The balanced cross-over design (BCD)

**Figure 2:**
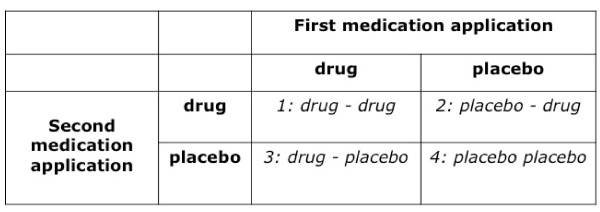
**The "balanced cross-over design" (BCD): **All subjects are told they participate in a double-blind cross-over design study and will receive both drug and placebo; this is true for groups 2 and 3, while in groups 1 and 4 they receive drug - drug and placebo - placebo, respectively.

In an attempt to overcome the serious limitations of the BPD, we designed another strategy that may account for some of the BDP limitations. In this case, subjects are divided into four groups, and all are told they participate in a conventional randomized double-blinded and placebo-controlled crossover trial, in which they will receive both the drug and the placebo at two different occasions in a randomized and double-blinded order. However, only group 2/3 will be exposed to drug and placebo in a balanced way, that is half the subjects will receive the drug first and the placebo at the second occasion, while the other half will receive first placebo and then the drug. Group 1 will receive drug - drug, and Group 4 will receive placebo - placebo instead (Figure 2).

In this case, Groups 2 and 3 represent the conventional drug trial assuming the "additive model" for drug and placebo response. In Group 1, the minimal value of both measures represents the "true" drug response, and the difference between both is the expectancy component of the drug response. In Group 4, the maximum value should represent the "true" placebo response; and the difference between both values should be the expectancy component of the placebo response. Comparing these expectancy effects between groups 1 and 4 allows to test whether the expectancy component of the placebo response is equal under drug and placebo condition - which is the assumption of the "additive model".

The BCD has one important methodological limitation: As with other crossover designs, interference of learning effects need to be kept in mind [20,21], and any adaptation or habituation between measurement 1 and measurement 2 should be minimized, e.g. by increasing the time interval between the two. Its ethical limitations (deception) are similar to those of the BPD with the exception, that subjects may receive a drug twice but expect it to receive only once - any risk involved in such a repetition of drug application would exclude the BCD from use, and it can only be used in patients when the deception is authorized [43].

To our knowledge, this design has not been used so far in any research, whether related to drug or to the placebo response.

### "Hidden treatment" according to Benedetti

While most designs and attempts to modify the response to a drug and explore the placebo response (as those discussed above) manipulate the information provided to the subjects, a few others, such as the "hidden treatment" paradigm [46] manipulate the timing of the drug action to identify whether and to what degree the placebo response can be elucidated.

Hidden treatment (HT) or covert treatment is an option that may be specifically useful for the test of drug effects in acute and highly symptomatic conditions such as with postoperative pain [47], anxiety, and motor dysfunction in Parkinson's disease [48,49]. It resembles some of the features of the "single subject trials" [31]. In case of HT, the patient receives a drug unnoticed in terms of timing and dosage, and the drug effect (or its missing action) can be determined independent of the patient's expectations. Benedetti and colleagues demonstrated that under these circumstances drugs commonly believed to have analgesic properties such as CCK-antagonists failed to show any anti-nociceptive effects [50]. Evidently, HT can only be applied with the patient being fully informed prior to the test that she/he will receive the drug in any case but will not be informed about when the drug is provided, otherwise the approach may raise other ethical concerns [51], especially with the test of novel compounds of unknown properties.

### The "delayed response" test

**Figure 3:**
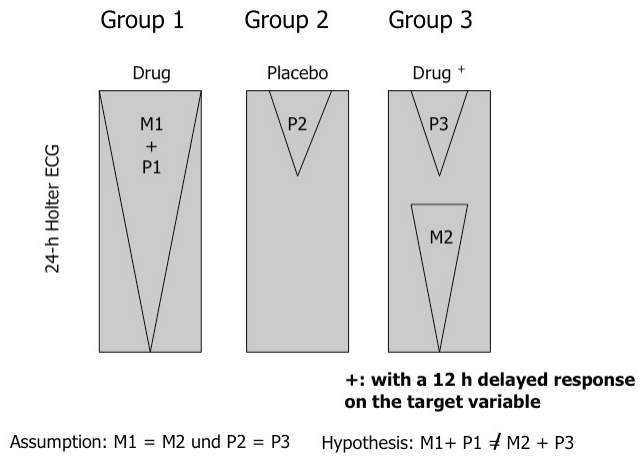
**The "delayed response test" (DRT) design: The "additive model" by Kirsch (2000) assumes that P1 = P2**. Under the further assumptions that M1 = M2 and P2 = P3, the hypothesis of the "additive model" is falsified if (M1+ P1 ≠ M2 + P3).

A very unique approach based on such an assumption that has - to our knowledge - never been explored in any placebo testing is the following: Assume that a drug can elicit its action at a predefined time point hours after ingestion, either via a coating technology [52] or a radio-transmitted mechanical capsule technology [53] for controlled release of the compound; such technology has been used in many clinical conditions such as diabetes, Alzheimer dementia and other conditions. In this case, a three-arm design (Figure 3) would allow a very elegant prove of the additive versus non-additive model of the placebo response.

All subjects receive the same information that they will receive either a drug or placebo in a double-blinded fashion, and no information is given about the timing of drug action; instead, a rational ("cover story") is provided for prolonged drug action monitoring, e.g. for 24 hours.

Group 1 will receive the drug with immediate action, group 2 the respective placebo. Group 3 receives the delayed response medication, e.g. with drug release after 12 hours. To confirm the "non-additive model" (P1 ≠ P2), M1 + P1 ≠ M2 + P3, with P3 = P2 and M1 = M2.

A variant of such a design that intended to elucidate the drug response in a clinical trial in Parkinson's Disease was recently described [32]. While this has never been used in a laboratory setup for assessment of the placebo response, preliminary approval of the underlying hypothesis may be drawn from clinical studies that have used the delayed release technology in the treatment of patients, e.g. in 5-ASA treatment of chronic inflammatory bowel diseases [54]: in these studies, the placebo response should be overall lower as compared to immediate release medication. Unfortunately, the number of placebo-controlled studies using such technology is so far rather low to allow a reliable metaanalytic comparison.

## Conclusions

Investigating the size and mechanisms of the placebo response in different medical conditions has relied on experimental procedures that simulate the double-blind randomized placebo-controlled design of clinical trials. However, as the conventional design is thought to elucidate drug rather than placebo actions, different methodological procedures are needed for the placebo response. In general, this can be done by either manipulation the information provided to the subjects, or by manipulation the timing of the drug applied. Two examples of each strategy are discussed: the "balanced placebo design" (BDP) and the "balanced cross-over design" (BCD) and their variants are based on false information provided, while the "hidden treatment" (HT) strategy and the "delayed response test" (DRT) are based on altering the timing of drug action. While most such approaches include deception of the subjects this limitation can be overcome by implementing "authorized deception".

## Competing interests

The authors declare that they have no competing interests.

## Authors' contributions

PE and SK developed the idea for this manuscript, reviewed the respective literature, discussed the potentials of alternatives to traditional trial designs, PE wrote the manuscript, and SZ helped writing the manuscript. All authors read and approved the final manuscript.

## Pre-publication history

The pre-publication history for this paper can be accessed here:

http://www.biomedcentral.com/1471-2288/11/90/prepub
